# Differential growth at the apical hook: all roads lead to auxin

**DOI:** 10.3389/fpls.2013.00441

**Published:** 2013-11-05

**Authors:** Mohamad Abbas, David Alabadí, Miguel A. Blázquez

**Affiliations:** Instituto de Biología Molecular y Celular de Plantas, Universitat Politècnica de València - Consejo Superior de Investigaciones CientíficasValencia, Spain

**Keywords:** apical hook, auxin, ethylene, gibberellin, hormone interaction, development

## Abstract

The apical hook is a developmentally regulated structure that appears in dicotyledonous seedlings when seeds germinate buried in the soil. It protects the shoot apical meristem and cotyledons from damage while the seedling is pushing upwards seeking for light, and it is formed by differential cell expansion between both sides of the upper part of the hypocotyl. Its apparent simplicity and the fact that it is dispensable when seedlings are grown *in vitro* have converted the apical hook in one of the favorite experimental models to study the regulation of differential growth. The involvement of hormones –especially auxin—in this process was manifested already in the early studies. Remarkably, a gradient of this hormone across the hook curvature is instrumental to complete its development, similar to what has been proposed for other processes involving the bending of an organ, such as tropic responses. In agreement with this, other hormones—mainly gibberellins and ethylene—and the light, regulate in a timely and interconnected manner the auxin gradient to promote hook development and its opening, respectively. Here, we review the latest findings obtained mainly with the apical hook of *Arabidopsis thaliana*, paying special attention to the molecular mechanisms for the cross-regulation between the different hormone signaling pathways that underlie this developmental process.

## Introduction

One of the developmental innovations during land plants evolution was the invention of skotomorphogenesis, most likely during the emergence of Angiosperms, forced by the need of seedlings to efficiently and safely grow toward the light when seed germination began to take place when buried in the soil (Wei et al., 1994). Seedlings that follow this developmental program show an etiolated appearance with a fast-growing and long embryonic stem (Fankhauser and Chory, 1997). The strategies followed by monocotyledonous and dicotyledonous seedlings to protect the shoot apical meristem while pushing through the soil are, however, different. While monocots have developed the coleoptile as a protective structure, the shoot apical meristem is protected by two small and folded cotyledons subtended at the tip of a hook-like structure in the upper part of the hypocotyl in most dicots. Remarkably, the presence of the apical hook has become key for successful emergence from the soil after seed germination, as seedlings lacking this structure have lost this vital ability (Harpham et al., 1991).

**Figure 1:**
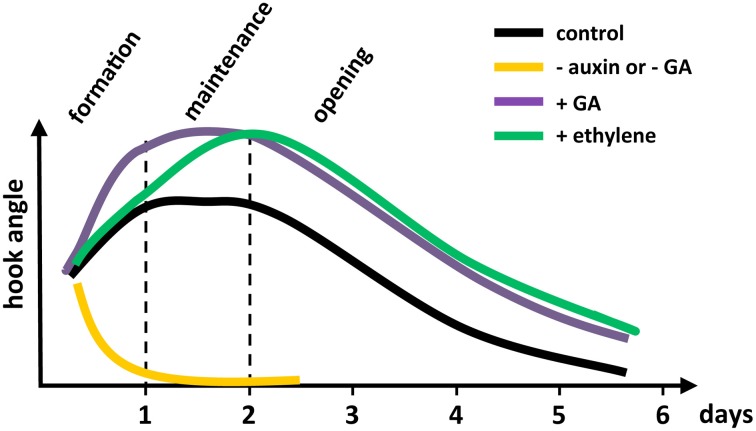
**Schematic illustration showing the dynamics of hormone actions in apical hook development in *Arabidopsis*.** The - auxin was produced by treating with the auxin transport inhibitor naphthylphthalamic acid (NPA); - GA, by GA biosynthesis inhibitor paclobutrazol; and + ethylene, by adding the immediate ethylene precursor, ACC. The + GA shows the GA action constitutively revealed by the use of a *dellaKO* mutant.

Time-lapsed imaging has allowed us to look at the dynamics of hook development in dark-grown *Arabidopsis* seedlings with an unprecedented precision, starting at seed germination (Vandenbussche et al., 2010; Zadnikova et al., 2010; Gallego-Bartolomé et al., 2011), supporting and extending previous studies (Raz and Ecker, 1999). When growing *in vitro*, apical hook development proceeds through three different phases (Figure 1; see the whole process of *Arabidopsis* hook development in Movie 1 available in Supplemental Material). The formation phase starts when the seedling emerges from the seed coat, and lasts about 24 h in which the hook reaches roughly 180°. This phase is followed by a maintenance phase, in which the seedling actively keeps its hook closed for another about 24 h while the hypocotyl rapidly elongates. Finally, seedlings enter the opening phase in which the hook starts to open, reaching angle zero 3 days later. As we will review in the following sections, this technique has served to precisely dissect the involvement of different hormone pathways in each phase of hook development, taking advantage of the fact that this structure is dispensable under conventional *in vitro* conditions.

How is the hook curvature achieved? The growth of an organ is the consequence, in the simplest view, of coordinated cell division and cell expansion. Thus, in principle both processes might occur differentially at opposite sides of the hypocotyl and contribute to generate the curvature. Early studies, however, showed that hook curvature is mainly caused by differential cell expansion between both sides at the upper part of the hypocotyl, in such a way that the side with the higher growth rate becomes the outer side of the hook (Silk and Erickson, 1978). Nonetheless, a small but significant contribution of differential cell division also occurs, being higher at the inner side of the hook and consequently leaving less space for cells to enlarge (Raz and Koornneef, 2001).

## An auxin gradient drives differential cell growth at the apical hook

Once established the cellular basis of the curvature, the next relevant question was about the driving force underlying the differential cell growth. It was known for many years that an organ, for example the stem or the root, can bend in response to tropic environmental stimuli, and that a differential cell elongation at opposite sides of the organ lays at the base of the response (Esmon et al., 2005). Importantly, this differential growth is driven by an asymmetrical distribution of the hormone auxin triggered by the tropic stimulus (Spalding, 2013), as the Cholodny-Went model proposed back in 1926 (Went, 1974). For instance, auxin accumulation is higher at the shaded than at the lit side of hypocotyls of etiolated *Brassica oleracea* seedlings exposed to unidirectional blue light, causing its elongation and thus bending toward light (Esmon et al., 2006).

Hence, does an auxin gradient drive the differential growth in the apical hook too? First hints came from physiological analyses in etiolated *Phaseolus vulgaris* seedlings, which showed preferred auxin accumulation at the inner side of the hook (Schwark and Schierle, 1992). Genetic confirmation for the involvement of auxin came later, when *Arabidopsis* mutants over-accumulating the active auxin indole-3-acetic acid (IAA) showed a *hookless* phenotype (Boerjan et al., 1995; Lehman et al., 1996; Zhao et al., 2001). The same was true for mutants with altered auxin response in the region where the hook should be (Lehman et al., 1996; Li et al., 2004). Indeed, staining of the auxin signaling marker *DR5::GUS* (see Glossary, Box 1), which usually stains the inner side of the hook in *Arabidopsis*, is lost in the latter mutants or after treatments with inhibitors of polar auxin transport that result also in *hookless* seedlings (Figure 1) (Friml et al., 2002; Li et al., 2004; Vandenbussche et al., 2010; Zadnikova et al., 2010; Gallego-Bartolomé et al., 2011; Willige et al., 2012). All these results pointed out that proper auxin distribution and response between both sides at the upper part of the hypocotyl are critical for the formation of the hook. It is important to remark that auxin signaling is enhanced at the side with restricted growth likely as a consequence of auxin accumulation, as occurs in the root after gravi-stimulation (Friml et al., 2002; Ottenschlager et al., 2003; Band et al., 2012), whereas the contrary occurs after gravi- and photo-stimulation in the shoot, as mentioned above. The preferential activation of specific ARF transcription factors that regulate growth negatively will cause the growth arrest in the inner side of the hook.

Box 1 **Glossary**

**ABCB1/ABCB19**

Membrane proteins that act as auxin efflux carriers, sending auxin out of the cell.

**ACOs**

Enzymes that catalyze the conversion of ACC into ethylene. It is assumed that their activity is not limiting. AtACO1: *Arabidopsis* ACO1. PsACO1: pea ACO1.

**ACSs**

The rate limiting enzymes in the ethylene biosynthesis pathway that convert SAM into ACC.

**ARFs**

Transcription factors that ultimately regulate gene expression in response to auxin. Play a positive role in the auxin signaling cascade. ARF: Auxin responsive factor.

**AUX/IAAs**

Negative regulators in the auxin signaling pathway that interact with and inactivate the ARFs in the absence of the hormone. Auxin triggers their degradation via the 26S proteosome.

**AUX/LAX**

Membrane proteins that transport auxin into the cell. LAX: Like AUX.

**DELLAs**

Transcriptional regulators that negatively regulate the GA signaling pathway. Mutant plants lacking DELLA activity have the GA signaling constitutively active.

***DR5::GUS***

Popular reporter whose activity is directly regulated by ARF transcription factors, and thus it reports the activity of the auxin signaling pathway.

***EBS::GUS***

Reporter whose activity is directly regulated by EIN3 and its paralogs. It is a reporter of ethylene signaling.

**EIN3**

Transcription factor that occupies a central place in the ethylene signaling pathway. Its activity is necessary to translate the ethylene signal into changes in gene expression.

**HLS1**

Putative acetyltransferase whose activity is critical for the formation of the apical hook. Its expression is induced jointly by GAs and ethylene, and repressed by light. HLS1: Hookless1.

**PIFs**

Transcription factors that promote elongation growth, among other processes, and regulated by DELLAs and light. PIFs: Phytochrome-interacting factors.

**PINs**

Membrane proteins that mediate auxin efflux.

**TAA1/TAR2**

Tryptophan aminotransferases that catalyze the first step in auxin biosynthesis. TAR: TAA-related.

**YUCs**

Flavin monooxygenases that catalize the second and last step in auxin biosynthesis.

**VAS1**

Methionine aminotransferase that reduces the amount of substrates of YUCs and ACSs, 3-IPA and SAM, respectively. Its activity therefore reduces the amounts of auxin and ethylene.

**WAG2**

Protein kinase whose activity likely regulates PIN localization in the cell. *WAG2* expression is induced by GAs through PIF5.

**Figure 2:**
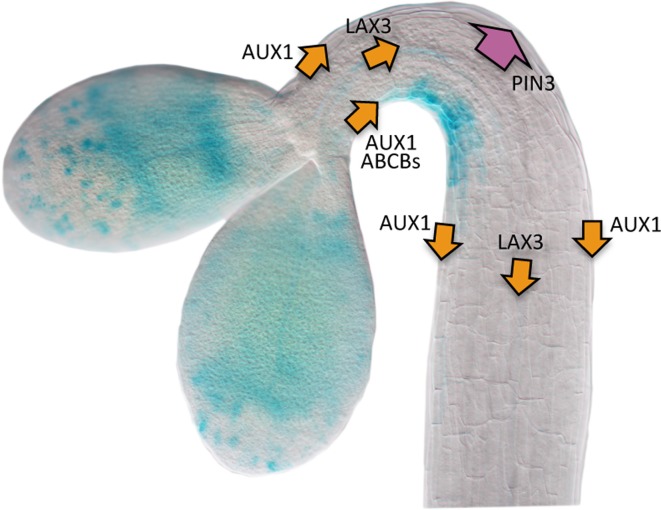
**Scheme of the transport machinery involved in the generation of the auxin gradient in the apical hook.** The auxin flow directed by influx and efflux carriers is represented by orange and purple arrows, respectively. The wide arrow representing PIN3 activity means that this carrier performs a major role driving auxin toward the outer side of the hook. The image represents a *DR5::GUS* seedling during the formation phase. Picture courtesy of Dr. Javier Gallego-Bartolomé.

Nowadays we have quite a good view of how the auxin gradient is formed (Figure 2). As mentioned above, treatment with inhibitors that block polar auxin transport causes seedlings to be *hookless* and, remarkably, enhances the *DR5::GUS* activity in cotyledons, suggesting that these act as a source of the hormone (Li et al., 2004; Vandenbussche et al., 2010; Zadnikova et al., 2010; Gallego-Bartolomé et al., 2011). Nonetheless, at least two evidences suggest that local auxin biosynthesis at the apical hook region might also contribute. First, *YUC1*, and *TAA1/WEI8* and *TAR2* genes (see Glossary, Box 1), encoding key enzymes that sequentially catalyze the two steps of the main auxin biosynthesis pathway (Mashiguchi et al., 2011; Stepanova et al., 2011; Won et al., 2011), are expressed at the apical hook region (Stepanova et al., 2008; Vandenbussche et al., 2010). And second, *wei8 tar2* and *yuc1/2/4/6* mutant seedlings are not able to form properly the apical hook (Stepanova et al., 2008, 2011; Vandenbussche et al., 2010).

In general, the expression of the auxin biosynthetic genes is not asymmetrical in the apical hook, and only during the opening phase *YUC1* becomes differentially expressed in the outer side (Vandenbussche et al., 2010), whereas *TAR2* expression is enhanced by ethylene specifically at the inner side during maintenance (Stepanova et al., 2008; Vandenbussche et al., 2010). This suggests that differential auxin biosynthesis is not a major determinant for the formation of the auxin gradient in the hook. Indeed, auxin transport is required not only to distribute in the hook the hormone coming down from cotyledons, but also the auxin synthesized at the hook itself. Despite some auxin may freely diffuse into the cells, most of it is transported actively by dedicated influx and efflux carriers, which are plasma membrane proteins that help auxin to move into and out of the cell, respectively (Spalding, 2013). The influx carriers in *Arabidopsis* are encoded by four genes (Peret et al., 2012), and among them *AUX1* and *LAX3* perform a major, additive role directing the auxin stream in the apical hook (see Glossary, Box 1) (Vandenbussche et al., 2010). In particular, AUX1 loads the cells of the apical part of the hook with auxin coming from cotyledons and shoot apical meristem and, together with LAX3, directs the auxin flow down the hypocotyl toward the root. AUX1 is localized mainly in epidermal cells at both sides of the hook and LAX3 in the vascular tissue, not showing in any case asymmetry. Therefore, the influx proteins participate in keeping the proper basipetal flow of auxin through the hook, whereas their contribution to the generation of the gradient is, if any, minor.

At least two types of membrane proteins act as auxin efflux carriers, the *PIN* gene family composed of eight members (Grunewald and Friml, 2010), and two members of the B-type ATP-binding cassette transporters, ABCB1 and ABCB19 (see Glossary, Box 1) (Noh et al., 2001). Genetic analysis has shown that both ABCB proteins are needed to proceed through hook development, since the double loss-of-function mutant *b1-1 b19-1* shows defects in hook formation and opening (Wu et al., 2010). Interestingly, ABCB19 is localized at the plasma membrane of epidermal cells at the inner side of the hook, likely mediating basipetal auxin flow through this side (Noh et al., 2001; Wu et al., 2010). Indeed, *DR5::GUS* activity disappears in *b1-1 b19-1* seedlings, whereas a strong signal is detected in plants over-expressing ectopically *ABCB19* (Wu et al., 2010). The scenario is more complicated when we look at the PINs. Genetics, combined with detailed kinematic analysis of hook development and confocal microscopy have underscored a prominent role for PIN3, distributing auxin both from the vascular tissue into the cortex and epidermis and through these tissues down the hypocotyl, and acting mainly at the outer side of the hook (Zadnikova et al., 2010). Nonetheless, PIN3 is aided by other transporters to properly distribute the hormone, namely PIN1 in the vascular cylinder (Zadnikova et al., 2010) and the inner side epidermis (Willige et al., 2012), and PIN4 and PIN7 in the cortex and epidermis of both sides (Zadnikova et al., 2010). In summary, the uniform upload of auxin by AUX1 and LAX3 into the upper hypocotyl, combined with the joint activity of PINs and the ABCB transporters may finally result in a higher auxin draining from the outer side of the hook and, consequently, accumulation at the inner side, thus generating the hormone gradient.

**Figure 3:**
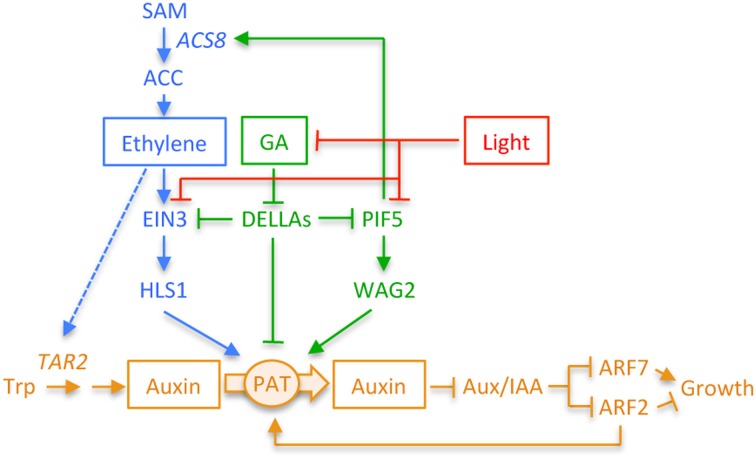
**Diagram depicting the interactions between GAs, ethylene, and light signaling pathways in *Arabidopsis* and how they modulate the auxin response in the apical hook.** Not all interactions take place simultaneously. The differential auxin response is required during the whole process of hook development, and it is modulated by GAs and/or ethylene depending on the phase. GAs are relevant during the formation phase, whereas the role of ethylene during this phase seems to be minor. The mechanism by which GAs control the auxin response during the formation phase is unknown, although it will likely involve *HLS1* regulation. Both GAs and ethylene are, however, important to prevent opening, and thus the interactions between both hormones will take place during maintenance. The signaling elements involved in the regulation of *TAR2* by ethylene are unknown (discontinuous blue line). Light may provoke hook opening at any stage of hook development. Bars and arrowheads indicate negative and positive effects, respectively. PAT, polar auxin transport.

The auxin gradient—measured as *DR5::GUS* activity—is established during the formation phase and disappears during hook opening (Vandenbussche et al., 2010; Zadnikova et al., 2010; Gallego-Bartolomé et al., 2011). The information contained in the gradient has to be interpreted by the signaling pathway to bring about the differential cell growth. As expected, several known elements of the auxin signaling pathway are involved in this response (Figure 3) (Chapman and Estelle, 2009). For instance, mutant seedlings defective in the four auxin F-box receptors lack an apical hook (Dharmasiri et al., 2005). The same phenotype is observed in plants expressing dominant, stable versions of the negative regulators in the signaling pathway, the Aux/IAA proteins SHY2/IAA3, BDL/IAA12 or IAA13 among others (see Glossary, Box 1) (Zadnikova et al., 2010), that are normally expressed at the inner side of the hook (De Grauwe et al., 2005; Zadnikova et al., 2010). The physiological importance of these local effects of auxin activity has been underscored by an elegant experiment in which the *axr3-1* dominant allele of an Aux/IAA gene was able to impair hook formation simply when its expression was specifically directed to the inner side of the hook (Vandenbussche et al., 2010). In agreement with this, loss-of-function mutants in some positive elements, such as the transcriptional activators NPH4/ARF7 and ARF19 (see Glossary, Box 1), present defects in hook development similar to the dominant mutations in *Aux/IAA* genes (Stowe-Evans et al., 1998; Harper et al., 2000; Zadnikova et al., 2010). The final output of the auxin signaling pathway on the hook is not mediated exclusively by ARFs that promote transcription. Genetic analyses demonstrate that the transcriptional repressors ARF1 and ARF2 act as negative regulators of hook development, as the double mutant *arf1 arf2* has a hook with an exaggerated curvature (Li et al., 2004). Interestingly, the transcriptional properties of the two types of ARFs, i.e., activation and repression, result in the promotion and repression of differential cell elongation, respectively (Stowe-Evans et al., 1998; Harper et al., 2000; Li et al., 2004; Okushima et al., 2005). Thus, the different auxin levels in both sides of the hook might simultaneously activate the two contrasting types of ARFs. Given that the ARFs seem to be expressed symmetrically in the hook (Li et al., 2004; Zadnikova et al., 2010), the ultimate effect on the elongation rate of both sides might respond to a different, spatially-driven sensitivity of each type of ARF to the activating properties of auxin, and/or to differences in the accumulation of the respective proteins (Figure 3).

As we have seen, auxin is instrumental for hook development. Nonetheless, it is not the only signal that contributes to this process (Alabadí et al., 2009). It has been known for many years that ethylene has a positive effect, since mutants affected in the ethylene biosynthetic or signaling pathways show alterations in hook development (Guzman and Ecker, 1990). Similarly, gibberellins (GAs) (Achard et al., 2003; Alabadí et al., 2004; Vriezen et al., 2004), and brassinosteroids (De Grauwe et al., 2005) also promote hook development. In the next two sections, we will review the latest results shedding light on how ethylene and GAs exert their action in hook development by modulating, at different levels, the auxin pathway.

## Ethylene regulates the auxin activity at different levels to promote hook development

The involvement of ethylene in the correct development of the apical hook was first suspected in the late 60s, when it was found that low concentrations of exogenous ethylene could inhibit hook opening in bean (Kang et al., 1967). That this effect was physiologically relevant was supported by the observation that the promotion of hook opening by light correlated with a decrease in ethylene production in bean (Kang et al., 1967) and in pea seedlings (Goeschl et al., 1967). More recently, genetic confirmation of the control of hook curvature by ethylene has been found through the evaluation of *Arabidopsis* mutants affected in ethylene biosynthesis and signaling. For instance, *eto1* and *eto2* ethylene overproducers (Guzman and Ecker, 1990), or the constitutive ethylene signaling mutant *ctr1* (Bleecker et al., 1988; Kieber et al., 1993) display an enhanced hook curvature, while *etr1* mutants defective in ethylene perception (Bleecker et al., 1988) or *ein2* mutants with a block in the ethylene signaling cascade are *hookless* (Guzman and Ecker, 1990). Real-time imaging of etiolated *Arabidopsis* seedlings has now showed that ethylene delays the transition between formation and maintenance phases, leading therefore to a hook with an exaggerated curvature (Figure 1) (Vandenbussche et al., 2010; Zadnikova et al., 2010; Gallego-Bartolomé et al., 2011).

An important aspect that has been intensively studied is the possibility that the differential cell growth rate in either side of the hook could be established by asymmetrical synthesis, perception, or signaling of ethylene in the top of the etiolated hypocotyl. Indeed, ethylene production is localized in the apical hook region of germinating seedlings (Goeschl et al., 1967), and it has been found to be unequally distributed in the apical hook cells of bean (Schwark and Bopp, 1993). However, there are contradictory results for the localization of ACC oxidases (see Glossary, Box 1) in hooks: while *PsACO1* mRNA was found preferentially in the inner side (Peck et al., 1998), *AtACO2* mRNA seems to accumulate in the outer, more rapidly elongating cells of the hook (Raz and Ecker, 1999). Despite this differential accumulation of *ACO* transcripts, it seems that the response to ethylene in the hook is not asymmetrical (Vandenbussche et al., 2010), as assessed using the primary ethylene response reporter *EBS::GUS* (see Glossary, Box 1) (Stepanova et al., 2007). In any case, ethylene application still shows a differential effect on either side of the hook, both in pea and in *Arabidopsis*, leading to the important question of how ethylene information is transformed into a differential growth effect.

Although ethylene has been proposed as an antagonist of auxin action for instance during lateral root formation and hypocotyl elongation (Muday et al., 2012), current evidences point in the direction of ethylene being a signal necessary for the establishment and maintenance of the auxin gradient that determines the differential growth rate between both sides of the hook (Figure 3) (Vandenbussche et al., 2010; Zadnikova et al., 2010). A genetic screen aimed at identifying regulatory elements for hook development induced by ethylene uncovered what seems to be a critical element that establishes asymmetry in the auxin across the hook. Loss-of-function mutations in *HOOKLESS1* (*HLS1*; see Glossary, Box 1) lacked an apical hook and completely suppressed the exaggerated curvature caused by ethylene application to etiolated *Arabidopsis* seedlings (Guzman and Ecker, 1990). *HLS1* encodes a putative N-acetyltransferase and its expression is positively regulated by ethylene, indicating that HLS1 mediates the ethylene-induced formation of the hook (Lehman et al., 1996). In fact, HLS1 is not only necessary, but also sufficient to promote hook formation, because its overexpression causes the formation of an enhanced hook curvature.

At least three pieces of evidence link HLS1 to the differential auxin response: (1) the lack of a proper gradient of auxin activity—measured as differential *DR5::GUS* expression—in the apical zone of the *hls1* mutant where the hook should have formed (Li et al., 2004); (2) the observation that auxin transport inhibitors phenocopy the suppression by *hls1* of the effects caused by exogenous ethylene (Lehman et al., 1996); and (3) the isolation of mutations in *AUXIN RESPONSE FACTOR2* (*ARF2*) as suppressors of *hls1* (Li et al., 2004). Moreover, sensitivity to ethylene is restricted to a time window of 2–3 days after germination (Raz and Ecker, 1999; Vandenbussche et al., 2010) strongly suggesting that the primary role of ethylene would be to help establish the auxin gradient and/or response after seedling emergence. The molecular mechanism by which ethylene performs this function is not completely clear yet. Given that ethylene still promotes hook formation in *HLS1* overexpressing seedlings (An et al., 2012), it is reasonable to think that ethylene impinges on more than one level at the auxin pathway for the generation of the auxin asymmetrical response.

In fact, several cross-regulatory points have been identified between both hormone pathways (Figure 3) (Stepanova et al., 2008; Vandenbussche et al., 2010; Zadnikova et al., 2010). On the one hand, ethylene enhances the auxin biosynthetic pathway in the inner side of the hook through local up-regulation of *TAR2* (see Glossary, Box 1), which is consistent with auxin acting downstream of ethylene, since IAA-treatments restore the hook in ethylene insensitive mutants (Vandenbussche et al., 2010). On the other hand, ethylene modulates to some degree the auxin transport in the hook, through the increase in both, the turnover of AUX1 in the inner side of the hook (Vandenbussche et al., 2010) and the preferential localization of PIN3 to the lateral side of cortex cells mainly at the outer side of the hook (Zadnikova et al., 2010). Moreover, it has been identified recently a protein, VAS1 (see Glossary, Box 1), that acts as a cross-regulatory point controlling the flow through the auxin and ethylene biosynthetic pathways in response to shade (Zheng et al., 2013). VAS1 prevents over accumulation of ethylene and auxin, thus preventing an exaggerated response to this environmental signal, and its expression overlaps with that of the *DR5::GUS* marker, at least at seedling stage and in flowers, suggesting that VAS1 activity contributes to the final outcome of auxin signaling. Thus, one can envision VAS1 acting in a similar way in the hook to control the proper accumulation of both hormones. In fact, it would be very interesting to study how hook development proceeds in *vas1* mutants to test this possibility.

In summary, ethylene seems to ensure the differential accumulation of auxin in the cells on the inner side of the hook, although it is not fully understood yet how HLS1 regulates the differential auxin response (Figure 3).

## Gibberellins modulate the auxin and ethylene action to regulate hook development

Time-lapsed imaging showed that GAs perform a prominent role during the formation and opening phases of hook development (Figure 1) (Gallego-Bartolomé et al., 2011). In particular, GAs are limiting during the formation phase, since mutant seedlings defective for the five DELLA proteins of *Arabidopsis* (see Glossary, Box 1), which are the negative regulators in the signaling pathway (Locascio et al., 2013) develop a hook with exaggerated curvature, whereas seedlings proceed directly to the opening phase when DELLA proteins over-accumulate.

Having seen in the previous sections the instrumental role of auxin for hook development, and how ethylene regulates it by modulating the auxin action, how do GAs fit within this scenario? Recent results identify several cross-regulatory points between GAs and the other two hormones (Figure 3) (Gallego-Bartolomé et al., 2011; An et al., 2012; Willige et al., 2012). The differential auxin response in the hook—assessed by *DR5::GUS* staining—depends on an active GAs (Gallego-Bartolomé et al., 2011). For instance, the asymmetrical response disappears during the formation phase when DELLAs over-accumulate due to inhibition of GA biosynthesis, whereas a GA treatment enhances it. Interestingly, this enhancement occurs only during maintenance and opening, suggesting that the GA activity is limiting to control the auxin response during these two phases and therefore the magnitude of the GA requirement is regulated developmentally. Asymmetry in GA signaling and response would explain the GA effect on auxin asymmetry. However, visualization of the DELLA protein RGA, whose activity is important for hook development (Alabadí et al., 2004) showed that this protein is evenly distributed through the hook (Vriezen et al., 2004), suggesting that asymmetry in GA signaling at the hook is minor, if any.

How do GAs control the auxin action in the hook region? First, GAs regulate the expression of auxin efflux carriers. Sustained expression of *PIN3* and *PIN7* requires active GAs, and in agreement with this, the *pin3 pin7* mutant does not show the enhanced curvature caused by exogenous GA (Gallego-Bartolomé et al., 2011). The molecular mechanism by which GAs regulate the expression of the transporters is currently unknown. Nonetheless, the requirement for GAs differs between both genes. Accumulation of DELLAs in the endodermis, but not in the epidermis, is enough to restrict *PIN3* expression to the vascular cylinder, while accumulation at any of both tissues results in *PIN7* repression. Given the prominent role of PIN3 in hook development, the effect on its expression seems to have consequences in the auxin transport, leading to the suppression of the differential response of *DR5::GUS* and very likely to hook opening.

Second, GAs control the expression of the *WAG2* gene (see Glossary, Box 1) (Willige et al., 2012). WAG2 is an AGC-type kinase that phosphorylates, at least *in vitro*, several PINs. *WAG2* is expressed preferentially at the inner side of the hook, where it prevents hook opening by helping to sustain proper asymmetry in the auxin response—likely through regulating PIN activity. Importantly, *WAG2* is induced by GAs through the transcription factor PIF5 (see Glossary, Box 1) (Willige et al., 2012), which participates in hook development (Khanna et al., 2007; Gallego-Bartolomé et al., 2011), and whose activity is inhibited upon interaction with DELLA proteins (Gallego-Bartolomé et al., 2011). Thus, it is very likely that GAs help to maintain auxin asymmetry at the hook by promoting DELLA degradation, which in turn allows PIF5 to enhance the expression of *WAG2* necessary to sustain proper PIN activity at the inner side of the hook. How *WAG2* expression is confined to the inner side of the hook is currently unknown.

As mentioned above, the GA and ethylene pathways interact in the hook region. Physiological analyses demonstrate that both hormones likely act independently of each other during hook formation, whereas they cooperate preventing opening (Gallego-Bartolomé et al., 2011). GAs contribute to maintain the threshold level of ethylene needed to proceed through hook development, mainly through maintenance and opening. Indeed, ethylene levels are higher in *dellaKO* mutants than in the wild type during these two phases (Gallego-Bartolomé et al., 2011). Remarkably, the expression of *ACS5/ETO2* and *ACS8* genes (see Glossary, Box 1), which encode key ethylene biosynthetic enzymes, is sustained by GAs. In particular, GAs promote DELLA degradation allowing PIF5 to bind to the promoter of *ACS8* and activating its expression (Gallego-Bartolomé et al., 2011), in a mechanism very similar to the regulation of *WAG2* by GAs (Willige et al., 2012). The contribution of DELLA-regulated ethylene biosynthesis to hook development may be, however, minor given that *dellaKO* mutants are mostly resistant to ethylene biosynthesis inhibitors (An et al., 2012).

In addition to regulating ethylene biosynthesis, GAs directly upregulate the expression of the ethylene-inducible gene *HLS1* (Gallego-Bartolomé et al., 2011; An et al., 2012). Time-lapsed imaging analyses show that there is a total coincidence in the timing of requirement of GAs and HLS1 activity for hook development (Gallego-Bartolomé et al., 2011), and that HLS1 is absolutely necessary for GAs to exert their role on this process (Gallego-Bartolomé et al., 2011; An et al., 2012). How do GAs regulate *HLS1*? The DELLA protein GAI directly downregulates the expression of *HLS1* (Gallego-Bartolomé et al., 2011), whereas EIN3 (see Glossary, Box 1) binds *in vivo* to the *HLS1* promoter in response to ethylene (An et al., 2012). Remarkably, An and colleagues (An et al., 2012) have shown that DELLAs are able to inactivate EIN3 upon physical interaction, thus providing a molecular mechanism for the interaction between both hormones to regulate, at least, the *HLS1* gene. This regulation might be relevant during the formation phase. However, it is not clear how relevant this regulation may be in normal situations, given that although GAs are limiting during this phase to determine the extent of hook curvature, they do not appear to be essential for *HLS1* expression or to establish the asymmetrical auxin response, assessed by *DR5::GUS* (Gallego-Bartolomé et al., 2011).

GAs and ethylene jointly prevent hook opening, and this is evident when both hormone pathways are fully active (Gallego-Bartolomé et al., 2011). This could be explained through the negative effect of DELLA on EIN3 activity. Nonetheless, genetic impairing of the activity of EIN3 and of its closest homologue EIL1 in a *dellaKO* background, does not result in *hookless* phenotype (An et al., 2012), indicating that GAs also act through additional transcription factors to prevent opening.

In summary, GA action on hook development is mediated by its effect on auxins, either directly or indirectly through their influence on ethylene (Figure 3). In this network, DELLA interaction with other transcription factors—PIF5 and EIN3—is crucial to bring about the GA control on ethylene and auxin. Nonetheless, further work is needed to fully understand the way GAs regulate hook development. For instance, our current knowledge does not explain the enhanced speed of hook formation of the *dellaKO* mutant.

## Light triggers hook opening

**Figure 4:**
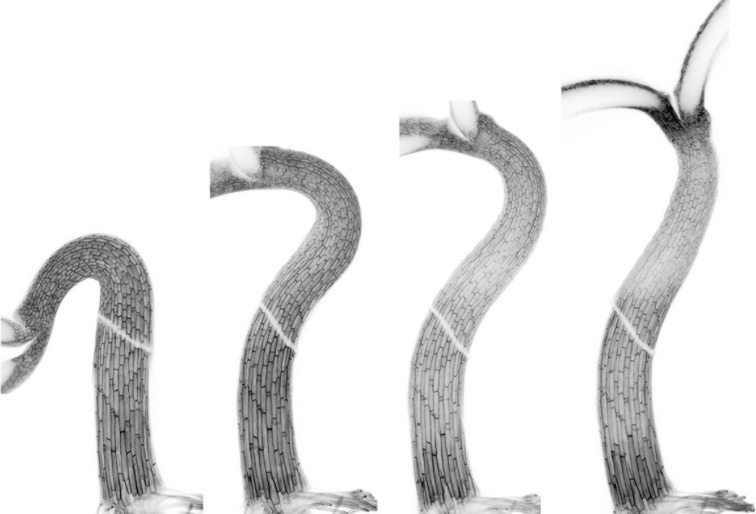
**High-resolution images of a single *Arabidopsis* seedling undergoing hook opening during de-etiolation.** From left to right: 2-day-old dark-grown seedling at the end of the maintenance phase; the same seedling 2, 4, and 8 h after illumination, respectively. The middle part of hypocotyls was removed—white oblique lines—to prepare the final images showing both the bottom and apical parts of hypocotyls. Pictures courtesy of Dr. Javier Gallego-Bartolomé.

Once the seedling emerges from the soil, the apical hook becomes dispensable and light triggers its opening, which is noticeable in less than an hour (Figure 4) (Liscum and Hangarter, 1993a; Miller et al., 2007; Wang et al., 2009; Wu et al., 2010). Light of different qualities trigger photomorphogenic responses, and among them hook opening is most sensitive to far-red and blue irradiances, whereas it is less sensitive to red under continuous irradiation (Liscum and Hangarter, 1993a). In a pulse irradiation, red light is most effective, its action being reversed by a subsequent far-red pulse. These provide the evidence that hook opening involves phytochrome actions, although cryptochrome is possible to partially contribute to continuous blue action (Liscum and Hangarter, 1993b). Accordingly, the phytochrome-regulated transcription factors PIFs are relevant modulators of this process (Figure 3) (Khanna et al., 2007; Gallego-Bartolomé et al., 2011; Kim et al., 2011; Willige et al., 2012).

It is reasonable to think that light impinges on the signaling network described in the previous sections to trigger opening of the hook. In fact, the auxin gradient, measured as *DR5::GUS* activity, disappears 4 h after the exposure of seedlings to light (Wu et al., 2010). The ABCB1 and ABCB19 auxin transporters might be targets of light signaling, since opening is delayed in seedlings that either lack both activities or express ectopically the ABCB19 protein (Wu et al., 2010). Nonetheless, it is still possible that these proteins' activity is not altered by light, and that the effect on the opening kinetics observed in the mutant lines is an indirect consequence of their defects in hook development, which are most apparent in the case of *b1-1 b19-1* seedlings (Wu et al., 2010). In fact, hooks eventually open and *DR5::GUS* activity decreases in plants that over-express ectopically ABCB19, suggesting that light signaling is able to overcome the activity of this transporter through alternative pathways.

One of the proteins that likely represents a major target of light signaling to control hook opening is HLS1. As mentioned in the previous sections, HLS1 activity constitutes a bottleneck for hook development, affecting auxin signaling (Lehman et al., 1996; Li et al., 2004). The way light signaling controls HLS1 seems to be at the transcriptional level, since the protein rapidly decreases upon illumination of etiolated seedlings, whereas it is stable for several days in the light when the *HLS1* gene is under a constitutive promoter (Li et al., 2004). As HLS1 decreases, there is a concomitant increase in ARF2 that will likely affect negatively the differential auxin response across the hook, causing its opening. *HLS1* gene transcription is up-regulated by GAs and ethylene (Lehman et al., 1996; Gallego-Bartolomé et al., 2011; An et al., 2012), as mentioned in the previous sections, in an EIN3-dependent manner (An et al., 2012). Therefore, it would not be surprising that light signaling down-regulates *HLS1* by impinging on ethylene and GAs (Figure 3). On the one side, EIN3 protein is destabilized by light (Zhong et al., 2009), and although the rate of EIN3 decrease is quite slow, it is reasonable to think that it might contribute to down-regulate *HLS1*. And on the other side, DELLA proteins accumulate in etiolated seedlings upon illumination (Achard et al., 2007), in parallel with the decrease in HLS1 (Li et al., 2004). Importantly, DELLAs might interact physically with EIN3 as they accumulate, and because of the interaction, EIN3's ability to bind to *HLS1* promoter is most likely compromised (An et al., 2012). Therefore, light might trigger hook opening by down-regulating *HLS1* through its dual, negative effect on EIN3. The extent to which the effect of light on *HLS1* is EIN3-dependent needs further investigations. The accumulation of DELLA proteins during de-etiolation surely has additional consequences on the signaling network. For instance, DELLAs will join light to regulate PIF5 negatively (Shen et al., 2007; Gallego-Bartolomé et al., 2011). Impairing of PIF5 activity should down-regulate *WAG2*, impinging on the activity of the PIN proteins (Willige et al., 2012), therefore contributing to promote hook opening. Despite these are likely targets of light signaling to open the hook, it will also impinge on the auxin gradient through other, unknown elements, given that light is still able to open the hook of *dellaKO* or ACC-treated seedlings.

## Perspectives

In summary, we have quite a fair understanding of the signaling network that regulates apical hook development. Nonetheless, new arising questions need to be answered in the next years to have a more complete and realistic view of this process. First, from a mechanistic point of view, we have to address the following issues: (i) determine how and when the polarity in the hypocotyl region where the hook will form is established. (ii) fill the gaps in the signaling network by identifying the transcription factors that mediate, for instance, GAs or ethylene effect on auxins. And (iii) identify the downstream, side-specific target genes of the network, in order to understand the actual processes that make the difference between both sides, as has been done in response to tropic stimulations (Esmon et al., 2006). For example, a transcriptional profiling of dissected hooks of etiolated soybean seedlings has rendered a few hook specific genes that are regulated by light during opening, and has permitted to assign a role in hook development to RPT2 (Li et al., 2011), a protein previously related to tropic responses (Sakai et al., 2000).

And second, from an ecological point of view, and besides having already shown that the apical hook confers a vital advantage to seedlings (Harpham et al., 1991), we have to try to understand how hook develops under natural conditions, ideally while seedlings are buried in the soil. All our knowledge has been built studying seedlings growing *in vitro*, owing to the dispensable nature of the apical hook under this condition. However, the relationships between the hormones identified and, more importantly, their relative importance might be very different in the wild, where other cues can take the lead directing hook development, for instance, soil type and compactness or oxygen availability.

## Conflict of interest statement

The authors declare that the research was conducted in the absence of any commercial or financial relationships that could be construed as a potential conflict of interest.

## Supplementary material

Movie 1 **Shows the *Arabidopsis* apical hook development process.** Apical hook development in *Arabidopsis* seedlings. Col-0 *Arabidopsis* wild type seeds were sown and germinated in regular medium in vertical plates. Images were taken every 45 min for 121 h with a camera Guppy F-146B (Allied Vision Technologies). Infra-red light was provided by an IR LED back-light emitting at 880 nm. Images were saved as a video in .avi format using the ImageJ software. One side of the squares in the background is 13 mm long.
